# Fabrication of untreated and silane-treated carboxylated cellulose nanocrystals and their reinforcement in natural rubber biocomposites

**DOI:** 10.1038/s41598-023-29531-x

**Published:** 2023-02-13

**Authors:** Narubeth Lorwanishpaisarn, Pongdhorn Sae-Oui, Sittipong Amnuaypanich, Chomsri Siriwong

**Affiliations:** 1 Materials Chemistry Research Center (MCRC), Department of Chemistry and Center of Excellence for Innovation in Chemistry (PERCH-CIC), Faculty of Science, Khon Kaen University, Khon Kaen, 40002 Thailand; 2 National Metal and Materials Technology Center (MTEC), National Science and Technology Development Agency (NSTDA), 114 Thailand Science Park, Pathum Thani, 12120 Thailand

**Keywords:** Materials chemistry, Polymer chemistry, Process chemistry, Surface chemistry

## Abstract

In this study, cellulose nanocrystal (CNC) was extracted from Napier grass stems and subsequently functionalized to carboxylated cellulose nanocrystal (XCNC) by using an environmentally friendly method, namely, the KMnO_4_/oxalic acid redox reaction. The XCNC was subsequently modified with triethoxyvinylsilane (TEVS), called VCNC, by using ultrasound irradiation. The characterization of the prepared XCNC and VCNC was performed. The needle-like shape of XCNC was observed with an average diameter and length of 11.5 and 156 nm, respectively. XCNC had a carboxyl content of about 1.21 mmol g^−1^. The silane treatment showed no significant effects on the diameter and length of XCNC. When incorporated into natural rubber (NR), both XCNC and VCNC showed very high reinforcement, as evidenced by the substantial increases in modulus and hardness of the biocomposites, even at very low filler loadings. However, due to the high polarity of XCNC, tensile strength was not significantly improved with increasing XCNC loading up to 2 phr, above which it decreased rapidly due to the filler agglomeration. For VCNC, the silane treatment reduced hydrophilicity and improved compatibility with NR. The highly reactive vinyl group on the VCNC’s surface also takes part in sulfur vulcanization, leading to the strong covalent linkages between rubber and VCNC. Consequently, VCNC showed better reinforcement than XCNC, as evidenced by the markedly higher tensile strength and modulus, when compared at an equal filler loading. This study demonstrates the achievement in the preparation of a highly reinforcing bio-filler (VCNC) for NR from Napier grass using an environmentally friendly method and followed by a quick and simple sonochemical method.

## Introduction

Napier grass (*Pennisetum purpureum*) is one of the most important fodder crops for livestock due to its low water and nutrient requirements for rapid growth. This crop is considered a highly cellulosic material because it is composed of approximately 46% cellulose and 34% hemicellulose^1–3^. Various techniques, i.e., chemical and/or mechanical treatments, have been used to extract cellulose by separating and removing lignin and hemicellulose^4–6^. Alkali treatment with concentrated sodium hydroxide (NaOH) followed by bleaching with sodium hypochlorite (NaClO_2_) is one of the most popular methods for obtaining high purity cellulose. The purified cellulose can then be turned into nano-structured cellulose via various chemical reactions, e.g., sulfate acid hydrolysis^7,8^, 2,2,6,6-tetramethylpyperidine-1-oxyl (TEMPO)-mediated oxidation^9^, and ammonium persulfate (APS) oxidation^10^. These methods have been widely used and suggested to be effective for the preparation of high purity cellulose with a high crystallinity of over 70%. However, the sulfate acid hydrolysis requires a massive amount of concentrated sulfuric acid, which has a negative impact on the environment. TEMPO-mediated oxidation is complicated and needs to be performed at a high pH value of 10–11 with several toxic reagents that can pollute the environment. APS oxidation also wastes a large amount of APS^11^. Due to the great concern about the environment, a new environmentally friendly method, namely, the potassium permanganate (KMnO_4_)/oxalic redox reaction, has been recently introduced^12,13^. Generally, KMnO_4_ in dilute sulfuric acid is used as a green oxidant because MnO_4_^−^ and Mn^3+^ can oxidize the amorphous component of cellulose. However, Mn^3+^ can be easily reduced to Mn^2+^ and, thus, the use of KMnO_4_ alone needs a relatively long reaction time. The addition of oxalic acid will turn Mn^3+^ to [Mn(C_2_O_4_^2−^)]^+^, which is a stronger oxidant, leading to a shorter reaction time and the formation of carboxylated cellulose nanocrystal (XCNC).

Cellulose nanocrystal (CNC) is one of the sustainable alternative fillers for natural rubber (NR). Due to its extremely small particle size and high stiffness, the NR biocomposites containing CNC generally possess a higher modulus and hardness. It has been previously uncovered that the addition of 2.5 wt% CNC, isolated from soy hulls, into NR considerably enhanced the tensile modulus of the composites (approximately 21 folds greater than that of the neat NR)^5^. However, the application of CNC in rubber reinforcement is still limited because the hydrophilicity of CNC, due to the abundancy of hydroxy groups, leads to not only the poor interaction between CNC and nonpolar NR, but also the high tendency of filler agglomeration, especially at high filler loadings. To improve the reinforcement of CNC, various surface modifications have been studied^14,15^. Silanization is one of the most promising surface modifications that can be achieved in aqueous media to prepare silane-treated nanocellulose. Various types of silane coupling agents have been employed^16–19^. Silane coupling agent is a bifunctional chemical that can react with both hydrophilic filler and hydrophobic polymer^20^. The surface modification of CNC with silane coupling agents can significantly improve the interaction between rubber and filler as well as the degree of filler dispersion in the rubber matrix^21^. Triethoxyvinylsilane (TEVS) is a bifunctional silane coupling agent that can be hydrolyzed to form silanol groups that can bond with hydroxy groups on the surface of hydrophilic fillers via a condensation reaction. Meanwhile, the vinyl group of TEVS can bond with NR during the sulfur vulcanization reaction^22^.

According to the strict legislation on environmental, health, and safety, green technologies for nano-filler preparation and surface modification are more desirable. In this study, XCNC was prepared from Napier grass stems through three chemical treatments, i.e., alkali treatment, bleaching, and KMnO_4_/oxalic acid redox reaction. The surface treatment of XCNC with TEVS was then carried out, to reduce the hydrophilicity of XCNC, by using a green synthesis technique, called ultrasound irradiation. This irradiation induces acoustic cavitation that causes the formation, growth, and collapse of gas bubbles. At the end of the collapse, localized hot spots are generated which increase temperature and pressure, resulting in a fast reaction rate (low reaction time) and improved reaction yield at relatively low input energy^23–25^. Both XCNC and TEVS-treated XCNC (VCNC) were subsequently characterized by various techniques before being added to NR. The properties of the filled NR biocomposites were then investigated and discussed.

## Materials and methods

Napier grass stems were supplied by the Bureau of Animal Nutrition Development, Khon Kaen, Thailand. Prevulcanized natural rubber (PNR) latex with approximately 60% dry rubber content (DRC) was obtained from VK industry, Nakhon Ratchasima, Thailand. Sodium hydroxide (NaOH) and potassium permanganate (KMnO_4_) were received from KemAus Co., Ltd., Australia. Hydrogen peroxide (H_2_O_2_), sodium chlorite (NaClO_2_), sulfuric acid (H_2_SO_4_), glacial acetic acid (CH_3_COOH), and hydrochloric acid (HCl) were received from Qrec Co., Ltd., New Zealand. Oxalic acid dihydrate (C_2_H_2_O_4_·2H_2_O), 97% triethoxyvinylsilane (TEVS), and AR-grade ethanol were obtained from Merck Co., Ltd., Germany.

### Preparation of XCNC

First, raw Napier grass stems were cut and placed in an oven at 60 °C for 24 h. The dried Napier grass stems were later milled by a knife mill, sieved through an 80-mesh screen, and subjected to the alkali treatment. The Napier grass sample (10 g) was soaked in 200 mL of 4 wt% NaOH solution under vigorous stirring for 24 h. The mixture was then filtered by using a Buchner funnel and repeatedly washed with hot distilled water until the pH of the filtrate was 7. The alkali-treated sample was then bleached using 200 mL of the bleaching solution, a mixed solution (1:1 v/v) of 1.7 wt% NaClO_2_ and acetate buffer, at 80 °C for 2 h, and filtered using a Buchner funnel. After three cycles of bleaching, the sample was rinsed with hot distilled water and filtered to obtain white cellulose powder. The preparation of XCNC was conducted through a KMnO_4_/oxalic acid redox reaction based on the literature with some modification^11^. The white cellulose powder was suspended in 300 mL of 1 M H_2_SO_4_ solution and mechanically stirred for 30 min in an ice bath before adding 10 g of KMnO_4_. Next, 5 g of oxalic acid was dissolved in 50 mL of 1 M H_2_SO_4_ solution, and then slowly added to the cellulose suspension, causing the color change from dark purple to dark brown. The suspension was stirred vigorously and refluxed at 50 °C for 8 h. The oxidation reaction was terminated when hydrogen peroxide (5 mL) was added dropwise into the dark brown suspension, turning it into a white suspension. The suspension was filtered and rinsed with deionized water to neutralize it. Finally, the filter cake was homogenized in deionized water to obtain an XCNC suspension with 2 wt% solid content using a homogenizer for 30 min.

### Preparation of VCNC

The XCNC suspension (50 g) was sonicated for 10 min before the modification. 64 mg of TEVS was dissolved in 50 mL of ethanol and then mixed with the pre-sonicated XCNC suspension. The pH of the mixture was adjusted with a drop of acetic acid and mechanically stirred for 10 min. The mixture was subsequently subjected to ultrasound irradiation for 30 min. A water bath (24 cm × 21 cm × 14 cm) was equipped with a 20 kHz and 200 W ultrasonic generator (AKHGZ, ACME ultrasonic tools, Thailand). This set-up allowed the sample to be held securely while the ultrasonic horn was turned on. The ultrasonic bath temperature was maintained at 30 °C by using a cooling system. The mixture was later centrifuged at 6000 rpm for 10 min. The obtained VCNC was washed with deionized water and ethanol at least three times to completely remove the un-reacted TEVS. The VCNC was finally re-dispersed in deionized water to obtain the VCNC suspension.

### Filler characterization

During the XCNC preparation, the percent yields of the products after the alkali treatment, the bleaching process, and the KMnO_4_/oxalic acid redox reaction were calculated using Eq. (1):

1 $$Yield\;(\% ) = \frac{{W_{1} }}{{W_{0} }} \times 100$$

where *W*_1_ is the mass of the dried sample from each step and *W*_0_ refers to the mass of Napier grass stems. The microstructure of the samples obtained from each preparation step was also explored by scanning electron microscopy (SEM model 1450VP, Leo, UK) with a gold sputtering coating machine. The morphology of XCNC was observed by transmission electron microscopy (TEM; FEI Technai G^2^ 20S Twin, Oregon, USA). The sample diluted with ethanol was dropped onto a 200-mesh carbon-coated copper grid and subsequently dried at ambient temperature before the examination. The average dimensions of XCNC were evaluated from 100 elements using ImageJ software. The density of XCNC was determined by a densitometer (MDS-300, Alfa Mirage, Japan). Determination of functional groups was performed by an attenuated total reflectance-Fourier transform infrared spectroscope (ATR-FTIR; Tensor 27, Bruker, Ettlingen, Germany). The carboxyl content of XCNC was measured by a conductometric titration method. Initially, 10 mg of XCNC was dispersed in 100 mL of 0.01 M hydrochloric acid (HCl). The suspension sample was mechanically stirred for 30 min prior to the titration with 0.01 M NaOH using a CDM210 conductivity meter equipped with a CDC866T electrode (Radiometer Analytical, France). The carboxyl content can be calculated using Eq. (2):

2 $${\text{Carboxyl}}\;{\text{content}} = \frac{{C_{{{\text{NaOH}}}} \times \Delta V}}{W}$$

where $C_{{\text{NaOH}}}$ is the concentration of NaOH (mol L^−1^), $\Delta V$ is the volume of titrant at the horizon section (mL), and $W$ is the weight of XCNC (mg). Crystalline structure and crystallinity index (*CrI*) were studied by X-ray diffractometer (XRD; Malvern Panalytical Empyrean, Royston, UK), using Cu-K_α_ radiation (λ = 0.15406 nm). According to Segal et al.^26^, the *CrI* was calculated using Eq. (3):

3 $$CrI(\% ) = \frac{{I_{200} - I_{{{\text{am}}}} }}{{I_{200} }} \times 100$$

where *I*_200_ refers to the peak intensity of the (200) crystallographic plane and *I*_am_ represents the peak intensity of the amorphous domain at 2θ = 18°. Thermal decomposition was studied by a Mettler Toledo thermogravimetric analyzer (TGA), Schwerzenbach, Switzerland. The temperature was scanned from 50 to 700 °C at a heating rate of 10 °C min^−1^ under a nitrogen atmosphere. X-ray photoelectron spectroscopy (XPS) was performed on a Kratos Axis Ultra DLD spectrometer (Manchester, UK). The survey spectra were used to calculate the atomic concentrations of oxygen (O), carbon (C), and silicon (Si). To determine the makeup of chemical bonds, high-resolution spectra of C 1*s*, O 1*s*, and Si 2*p* were analyzed. After the silane treatment, VCNC was characterized in a similar manner to XCNC before being added to NR.

### Preparation and testing of the NR biocomposites

Table 1 represents the compounding formulation of the PNR latex. Various amounts of the pre-sonicated XCNC and VCNC suspensions were added to the PNR latex in order to prepare the biocomposite containing various filler loadings ranging from 0 to 4 parts per hundred of rubber (phr).

**Table 1:** Formulation of the pre-vulcanized natural rubber (PNR) latex.

Ingredients	Amount (phr)
NR latex	100
Sulfur dispersion @ 50% w/w	1.0
Zinc oxide (ZnO) dispersion @ 50% w/w	1.0
Zinc diethyl dithiocarbamate (ZDEC) dispersion @ 50% w/w	0.5

The mixtures were stirred at 25 °C for 1 h using a magnetic stirrer with a speed of 250 rounds per minute (rpm). The homogenous mixtures were cast on a Petri dish and left at ambient temperature until the dried biocomposite sheets with approximately 2 mm thickness were obtained. The biocomposite sheets were later placed in an oven at 100 °C for 1 h prior to testing. The tensile test was carried out using a universal testing machine (UTM; INSTRON 5567A, Norwood, Massachusetts, USA). The sheets were cut into five dumbbell specimens (Type 1) and tested according to ISO 37. The tensile fracture surfaces of the specimens were coated by the gold sputtering machine before the SEM observation. Hardness was determined using a Shore A durometer (Wallace Instruments, H17A, UK) according to ISO 48-4. Five measurements of hardness at different positions on the test specimen were made. The degree of crosslinking was evaluated by an equilibrium swelling test. Three test specimens (1.0 cm × 1.0 cm × 0.2 cm) were prepared, weighed (*W*_d_), and submerged in toluene for 5 days at room temperature. The swollen specimens were then taken out of the toluene, immediately wiped with filter paper, and weighed (*W*_s_). Finally, the swelling degree can be calculated by Eq. (4).

4 $${\text{Swelling}}\;{\text{degree}}\; {{(\% )}} = \left( {\frac{{W_{{\text{s}}} - W_{{\text{d}}} }}{{W_{{\text{d}}} }}} \right) \times 100$$

For mechanical property and swelling tests, the average values are reported together with their standard deviation (SD). Morphology of the vulcanizates was studied by SEM (LEO/ZEISS 1450VP, UK). The tensile fractured surfaces were sputtered with a thin layer of gold prior to the examination. In addition, the degree of filler dispersion was also determined by TEM (FEI Technai G2 20S Twin, Oregon, USA). The ultrathin films were prepared by a Leica cryo-ultramicrotome at − 130 °C.

### Ethics declarations

The collection and experimental research on Napier grass comply with the Plant Variety Protection Act, Thailand (1999).

## Results and discussion

### Preparation and characterization of XCNC

To extract XCNC, Napier grass stems were subjected to three successive processes, *i.e*., alkali treatment, bleaching process, and KMnO_4_/oxalic acid redox reaction. The percent yields of these processes were 51.1%, 37.6%, and 33.3%, with regard to the original weight of the dried Napier grass stems. The density of XCNC was 1.42 ± 0.05 g cm^−3^.

**Figure 1:**
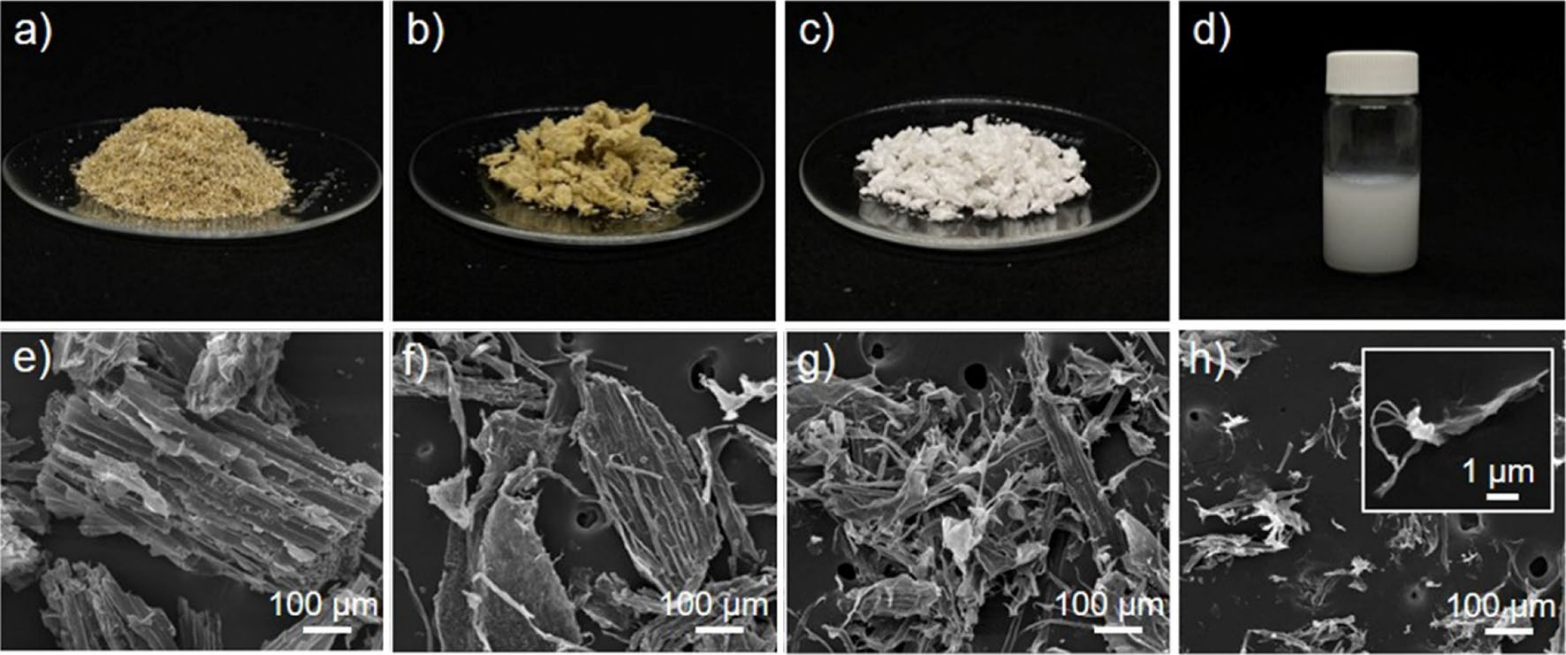
Photographs and SEM images; (**a**, **e**) raw Napier grass stems, (**b**, **f**) alkali-treated sample, (**c**, **g**) bleached sample, and (**d**, **h**) XCNC suspension.

**Figure 2:**
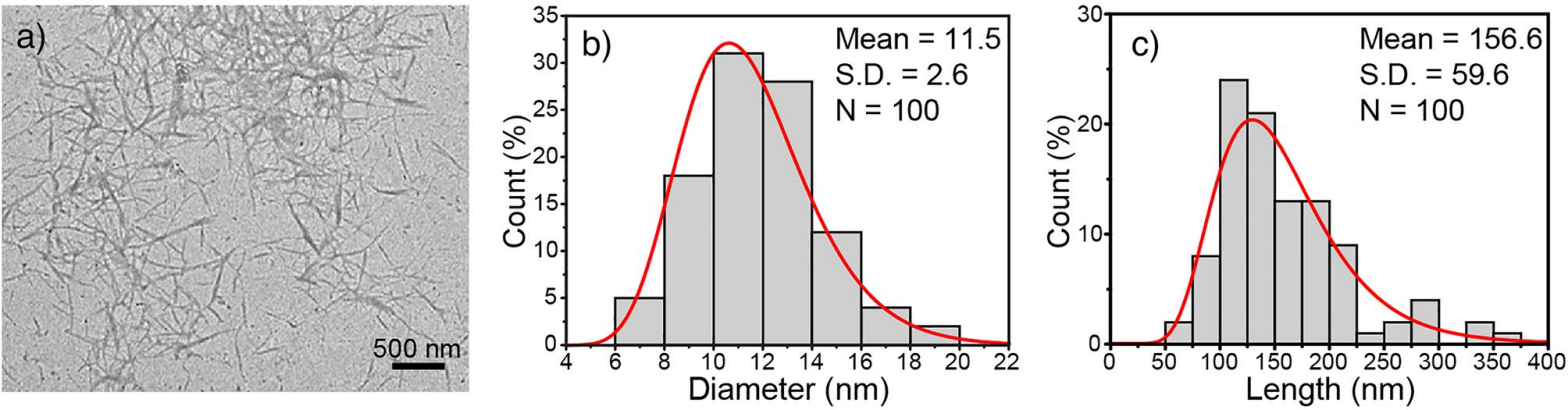
Morphology and size distribution of XCNC; (**a**) TEM image, (**b**) diameter distribution, and (**c**) length distribution.

The appearance of fiber products after each process is presented in Fig. 1. The raw, ground Napier grass stem was a brown powder with a very large particle size, as shown in Fig. 1a and 1e. The large particles consist of lignin, pectin, hemicellulose, and other non-cellulosic impurities that bond cellulose fibers to form such a huge structure. The alkali-treated sample showed a slightly brighter color (see Fig. 1b) because of the partial elimination of pectin, lignin, and hemicellulose. In addition, the smaller particle size was observed (Fig. 1f) due to the defibrillation. After the bleaching process which further removed the cementitious materials such as lignin and hemicellulose, the fiber turned white as shown in Fig. 1c and the fiber particles became smaller, see Fig. 1g, because such a removal leads to greater defibrillation. It has been reported that the alkalization and bleaching treatments could break the alkali-labile linkages, particularly ester and ether linkages, between lignin and other carbohydrates or between lignin monomers, resulting in the defibrillation of some parts of fibers^27,28^. After the fiber reacted with KMnO_4_ and oxalic acid, a white XCNC suspension was obtained, as depicted in Fig. 1d. When the XCNC suspension was freeze-dried and the obtained power was examined under SEM, the result shown in Fig. 1h reveals the existence of both micro-sized and nano-sized XCNC. The micro-sized fibers observed by SEM is thought to arise from the partial agglomeration of XCNC due to the strong hydrogen bonds among the abundant hydroxyl groups in the XCNC structure after drying^29^. Similar observation has previously been reported^7^. However, the TEM image in Fig. 2a, which was taken directly from the diluted XCNC suspension, shows that the particle size of XCNC was at the nanoscale level. Figure 2b and 2c exhibit respectively the diameter and length distributions of XCNC as measured by the ImageJ software. The diameter ranged from 6 to 19 nm, while the length was between 55 and 371 nm. The average diameter and length of the XCNC were 11.5 ± 2.6 nm and 156.6 ± 59.6 nm, respectively.

**Figure 3:**
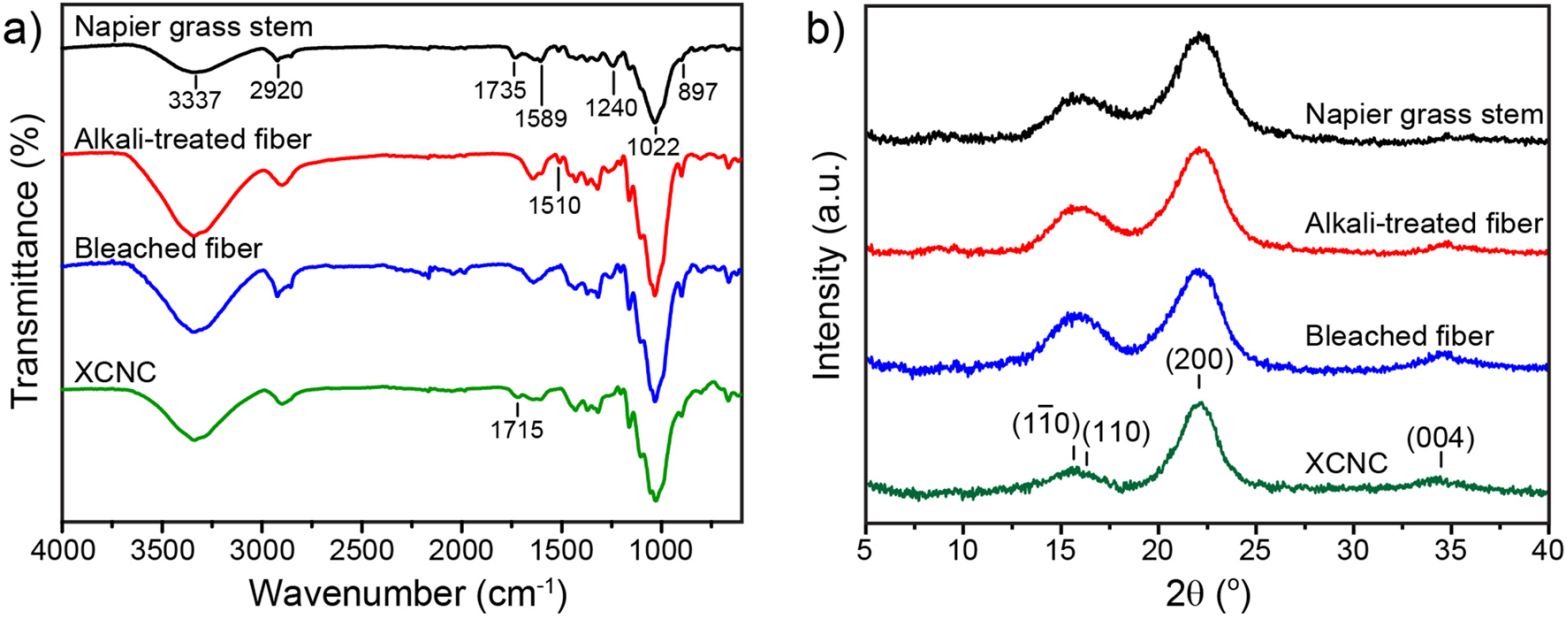
FTIR results (**a**) and XRD patterns (**b**) of the samples during the XCNC preparation.

The changes in chemical functional groups through the purification processes were investigated by FTIR. Figure 3a reveals the FTIR spectra of the samples obtained from each stage. Owing to the presence of cellulose, hemicellulose, lignin, and pectin, Napier grass stems possess many functional groups such as aromatics, alcohols, ketones, and esters. The peaks at 3337 cm^−1^ and 2920 cm^−1^ are associated, respectively, with the –OH and C–H stretching in lignin and other cellulosic components such as cellulose and hemicellulose. The vibration peak at 1735 cm^−1^ represents the stretching vibration of C=O in lignin and hemicellulose. The peak at 1589 cm^−1^ represents the C=C stretching vibration in the aromatic ring of lignin. The peak at 1240 cm^−1^ belongs to the C–O out-of-plane stretching vibration of the guaiacyl unit in lignin. The absorption peak at 1022 cm^−1^ is due to the asymmetric stretching vibration of C–O–C in cellulose and hemicellulose^30^. Moreover, the peak observed at 897 cm^−1^ is attributed to the *β*-glycosidic linkages between glucoses in cellulose^31^. After the alkali treatment, the absorption peaks at 1735 and 1240 cm^−1^ are considerably reduced due to the reduction in hemicellulose and lignin contents. During the treatment, sodium hydroxide can break hydrogen bonds between cellulose and these cementing materials and, thus, partially remove them from the sample. It can be noticed that the peaks at 897 and 1022 cm^−1^ are more pronounced after the alkali treatment, indicating a greater proportion of cellulose in the sample. After the bleaching process, the characteristic peaks of lignin at 1240 and 1510 cm^−1^ almost disappeared because of the further removal of lignin, which corresponded to the color change from yellowish to white. The FTIR spectrum of the sample after the redox reaction was similar to that of the bleached sample, except that a small additional peak was observed at 1715 cm^−1^, indicating the existence of carboxyl groups. By using the conductometric titration method, the carboxyl content of XCNC could be calculated and found to be 1.21 ± 0.06 mmol g^−1^ which is in the same range as previously reported in other publications^11,12^.

XRD patterns of the samples, shown in Fig. 3b, reveal the characteristic peaks at 2θ of 15.5°, 16.3°, 22.2°, and 34.6° corresponding to the cellulose I crystallographic planes ($1\overline{1}0$), (110), (200) and (004), respectively^32,33^. Owing to the progressive elimination of amorphous hemicellulose and lignin after each preparation step, the crystallinity index (*CrI*) values tended to increase, i.e., they were 57.5%, 62.0%, 62.3%, and 73.3% for Napier grass stem, alkali-treated specimen, bleached specimen, and XCNC, respectively. The increase in *CrI* values along the preparation step has previously been reported^7^.

**Figure 4:**
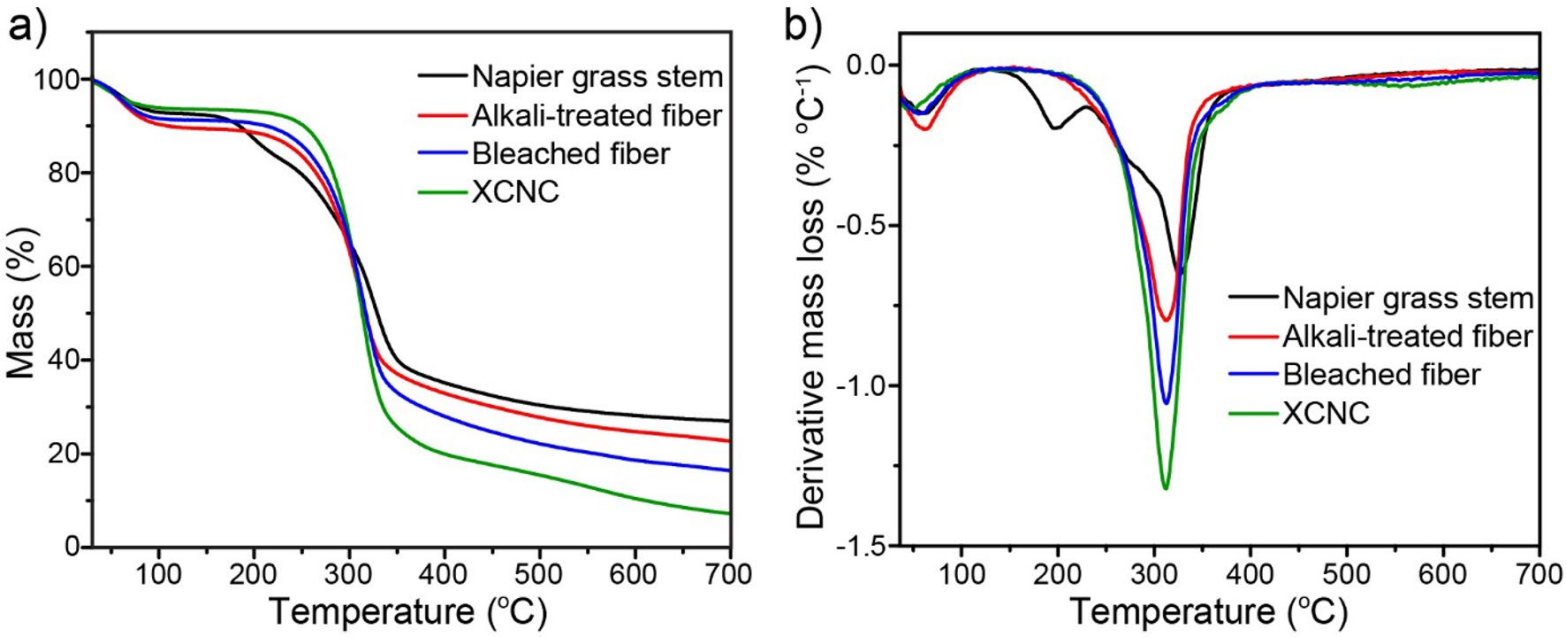
TGA results of the samples after each preparation process; (**a**) TG curves and (**b**) DTG curves.

Figure 4a and 4b show thermogravimetric (TG) curves and their derivative (DTG) curves of Napier grass stem, alkali-treated sample, bleached sample, and XCNC, respectively. The initial mass loss at a temperature below 100 °C, found in all samples, is attributed to the evaporation of water. For raw Napier grass stem, multiple decomposition stages occurred after 200 °C due to its multicomponent such as hemicellulose, lignin, and cellulose^34,35^. The initial decomposition was found at 200–250 °C, which is attributed to the depolymerization of hemicellulose^36^. The following decomposition, found at a relatively wide temperature range of 250–450 °C, is due to the overlap of the decomposition temperature of cellulose (250–400 °C) and that of lignin which rapidly decomposes at the temperature between 250 and 450 °C above which it gradually decomposes^37^. After the alkali treatment, the decomposition peak of hemicellulose (see Fig. 4b) disappears, implying the complete elimination of hemicellulose. After the bleaching process, lignin was mostly eliminated, leading to the shaper and narrower DTG peak (315 °C). After the redox reaction, XCNC shows the same decomposition characteristics as the bleached sample. However, compared with the bleached sample, XCNC possessed a higher percent mass loss at the temperature between 250 and 400 °C indicating the greater proportion of cellulose in the XCNC sample. At 700 °C, the carbon residue contents of Napier grass, alkali-treated fiber, bleached fiber, and XCNC were 27%, 25%, 16%, and 8%, respectively. The continuous reduction in carbon residue content is in good accordance with the progressive reduction in the hemicellulose and other non-cellulosic components after each treatment because it has previously been reported that both lignin and hemicellulose exhibit relatively high carbon residue content after the pyrolysis due to their high aromatic content^37^.

### Characterization of VCNC

**Figure 5:**
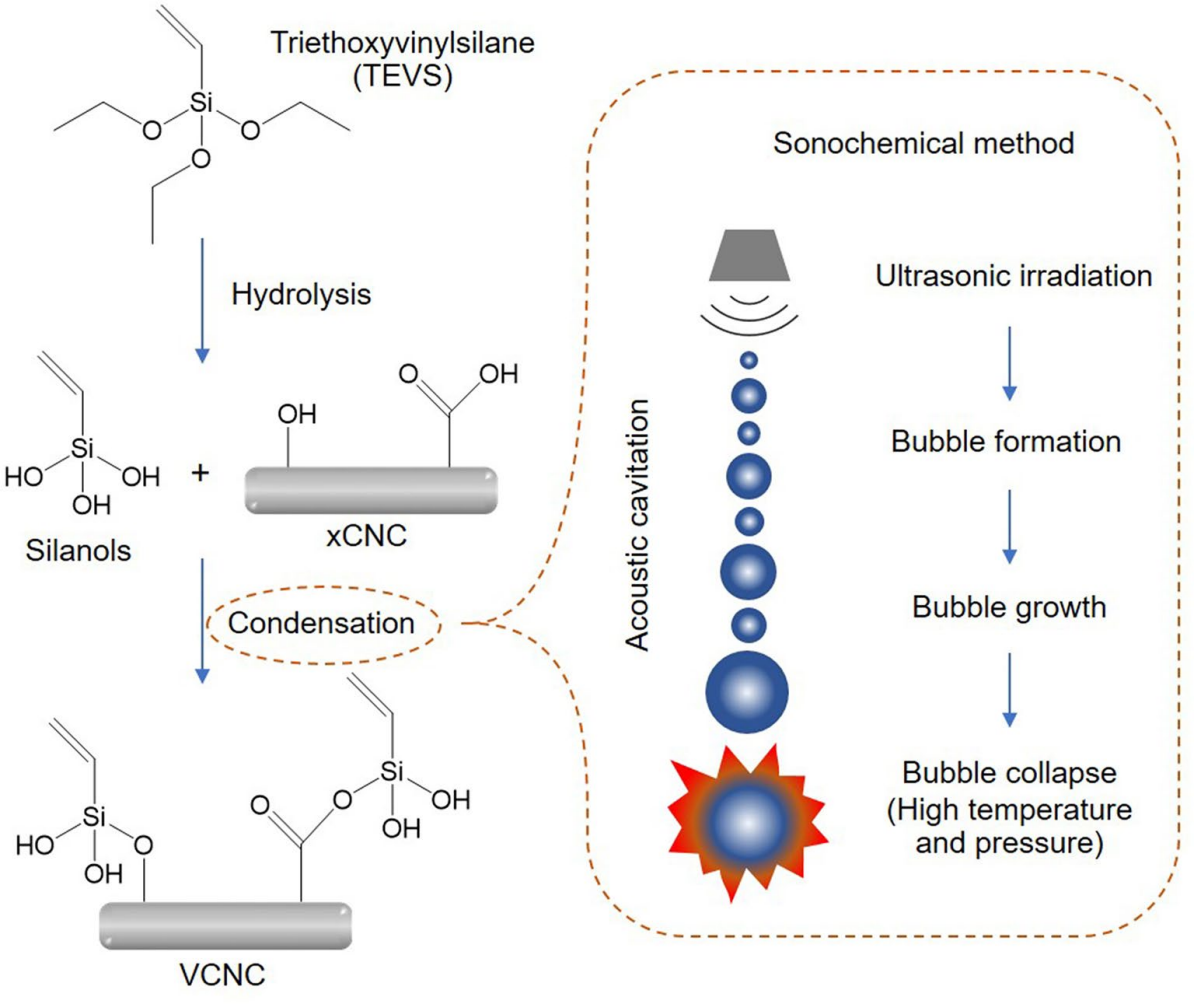
Proposed mechanism for surface modification of XCNC with TEVS through a sonochemical method.

After the ethoxy groups of TEVS were hydrolyzed to silanol groups under acid-catalyzed conditions, the condensation reaction between the silanol groups of TEVS and the carboxyl and hydroxy groups on the XCNC’s surface was carried out using an efficient, eco-friendly sonochemical method. The ultrasound irradiation generates a large number of cavitation bubbles, which grow and collapse. This phenomenon occurs over many cycles, leading to very high temperature and pressure that facilitate the condensation reaction^38,39^. The proposed mechanism for the VCNC preparation is shown in Fig. 5.

**Figure 6:**
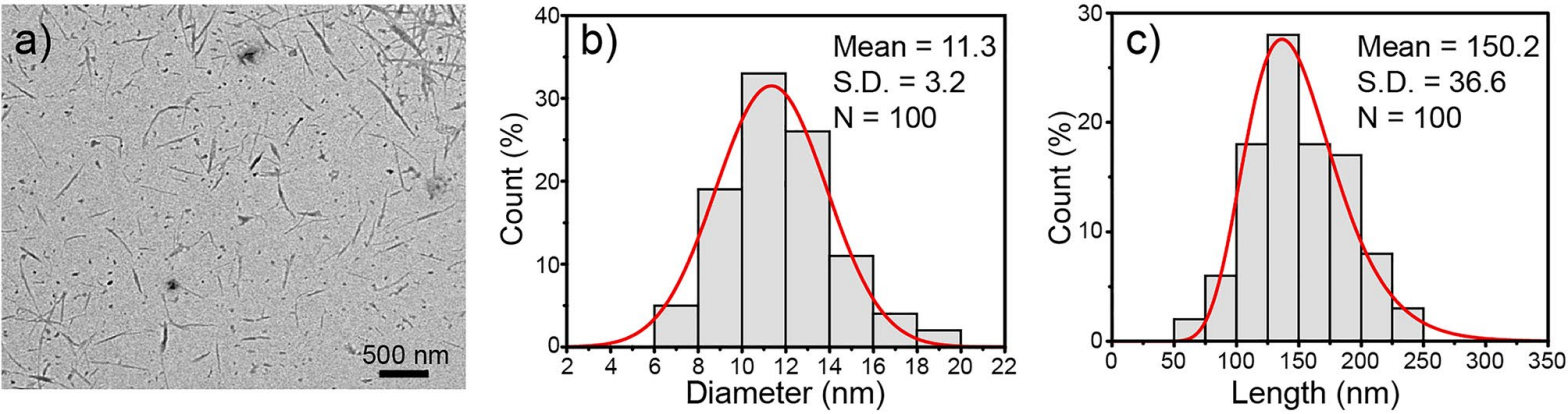
Morphology and dimensions of VCNC; (**a**) TEM, (**b**) diameter distribution, and (**c**) length distribution.

Figure 6 represents the TEM image and dimensions of the VCNC. Similar to the XCNC, the needle-like shape, with a very high aspect ratio, of VCNC was observed. The results clearly show that VCNC particles were well dispersed in aqueous media with relatively large length and diameter distributions. The diameter ranged from 6 to 19 nm, while the length was between 50 and 250 nm. The average diameter and length of VCNC were 11.3 ± 3.2 and 150.2 ± 36.6 nm, respectively. Compared with the results in Fig. 2, the results indicate that the silane treatment does not have a significant influence on the diameter and length of XCNC.

**Figure 7:**
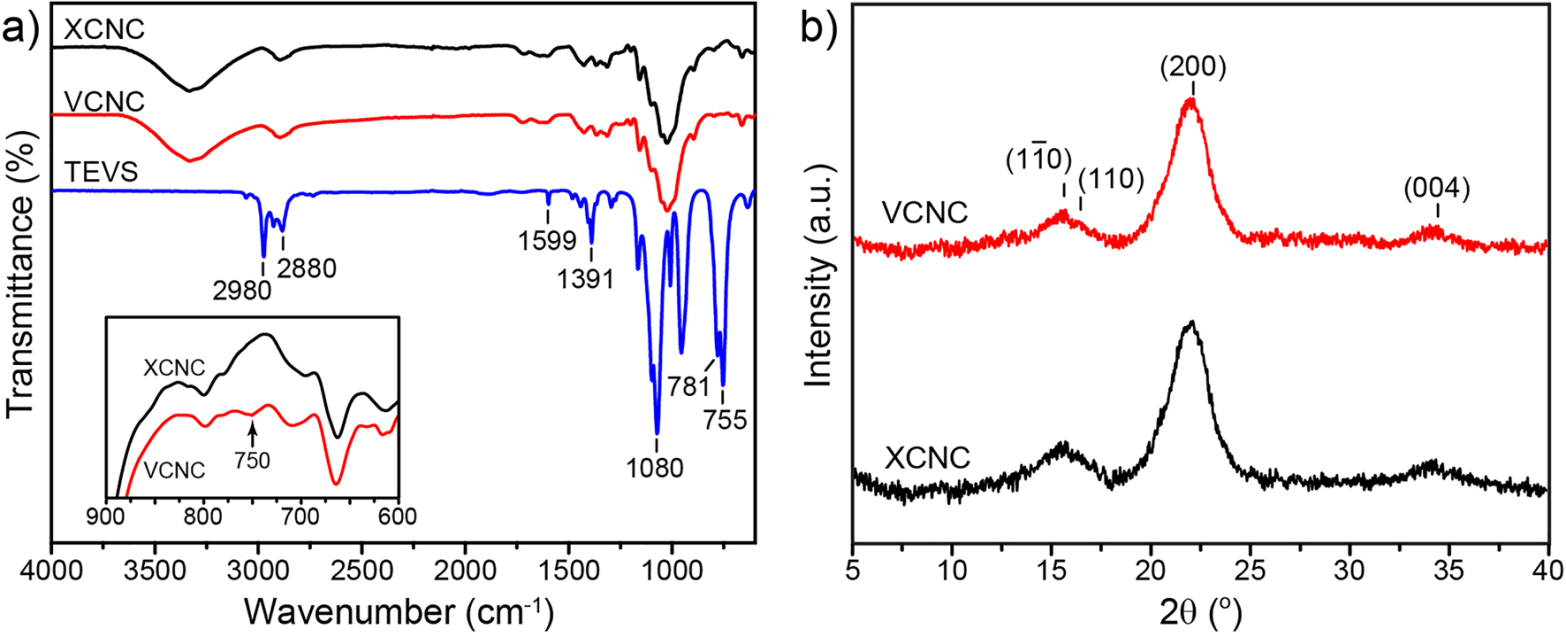
FTIR spectrum (**a**) and XRD results (**b**) of the XCNC and the VCNC.

Figure 7a displays the FTIR spectra of TEVS, XCNC, and VCNC. For TEVS, the spectrum shows peaks at 1599 and 1391 cm^−1^ assigned to the stretching vibration and in-plane deformation vibration of the vinyl group (–CH=CH_2_), respectively. The peaks at 2980 and 2880 cm^−1^ are attributed to CH_2_ and CH_3_ asymmetric and symmetric stretching vibrations in the aliphatic chains of TEVS. The strong peak at 1080 cm^−1^ belongs to the Si–O–C stretching vibration. Additionally, the peaks at 781 and 755 cm^−1^ are assigned to the symmetrical stretching vibration of Si–C. The characteristic peaks of XCNC have been identified previously (see Fig. 3). Apparently, the spectrum of VCNC is almost identical to that of XCNC, except that a small additional peak was found at 750 cm^−1^ implying the accomplished grafting of TEVS on the XCNC’s surface. However, the peak of Si–O–C stretching vibration cannot be used to confirm the attachment of TEVS on the XCNC surface because it is overlapped with the C–O–C broad peak of cellulose. The unsignificant change in the FTIR spectrum of cellulose after the silane treatment has also previously been mentioned^7,40^. A measurement of carboxyl content after the silane treatment was also carried out. The results reveal that, after the silane treatment, the carboxyl content declined from 1.21 ± 0.06 mmol g^−1^ to 0.79 ± 0.10 mmol g^−1^, implying a partial replacement of carboxyl groups by the silane component via condensation reaction^41^.

Figure 7b shows the XRD results of XCNC and VCNC. Obviously, both XCNC and VCNC possess the same diffraction pattern, showing peaks at 15.5°, 16.3°, 22.2°, and 34.6° 2θ reflections that are assigned to the ($1\overline{1}0$), (110), (200), and (004) crystallographic planes of cellulose structure I, respectively. The results clearly show that the silane treatment does not influence the crystal structure of XCNC. However, the *CrI* value after the treatment slightly declined from 73.3 to 70.3%. Similar findings have previously been uncovered^33,42,43^.

**Figure 8:**
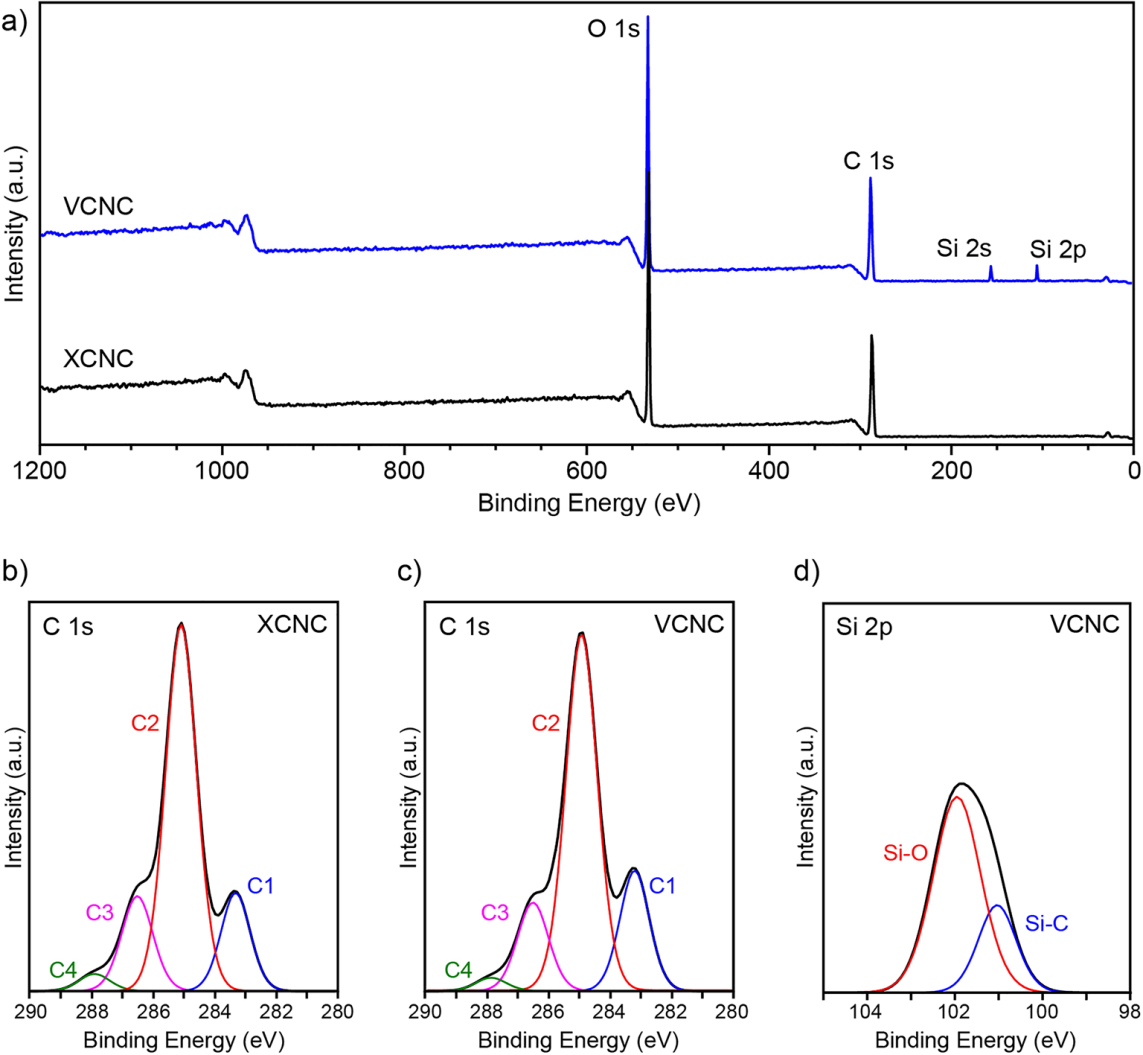
XPS spectra; (**a**) survey spectra of XCNC and VCNC, (**b**) high-resolution spectra of C 1*s* of XCNC, (**c**) high-resolution spectra of C 1*s* of VCNC, and (**d**) high-resolution spectra of Si 2*p* of VCNC.

The success of surface modification was also evaluated by XPS, as shown in Fig. 8. It can be clearly seen from the survey XPS spectra, shown in Fig. 8a, that XCNC showed only two main peaks of O 1*s* (at 531.0 eV) and C 1*s* (at 285.0 eV), while VCNC contained two additional peaks at 151.0 eV and 101.0 eV, which belong to Si 2*s* and Si 2*p*, respectively. The presence of these two peaks suggests the success of silane grafting on the XCNC surface. At higher resolution, the C 1*s* peak of XCNC can be deconvoluted into four peaks, as demonstrated in Fig. 8b. The C1 peak at a binding energy of 283.3 eV is attributed to the C–C, C–H, and C–Si bonds. The C2 peak at 285.0 eV belongs to the C–O bond. The C3 peak at 286.5 eV arises from the O–C–O bond. The C4 peak at 287.9 eV corresponds to the O–C=O bond. Similar to XCNC, the C 1*s* peak of VCNC can also be separated into four peaks, as shown in Fig. 8c. However, it can be observed that VCNC showed a slightly higher intensity of the C1 peak, in conjunction with a slightly lower intensity of the C2 peak as compared with XCNC. This can be explained by the existence of the vinyl silane in VCNC, which results in an increase in the C–C, C–H, and C–Si proportions in association with the simultaneous reduction in the C–O proportion. The degree of silanization is closely correlated with this intensity change.

The high-resolution Si 2*p* spectra of VCNC can also be deconvoluted into two peaks, as shown in Fig. 8d. The peaks at 101.2 eV and 102.0 eV are attributed to the Si–C and Si–O bonds, respectively. Again, it can be observed that the peak intensity of Si–O is higher than that of Si–C, indicating the effective grafting of silane onto the XCNC surface^44^.

**Figure 9:**
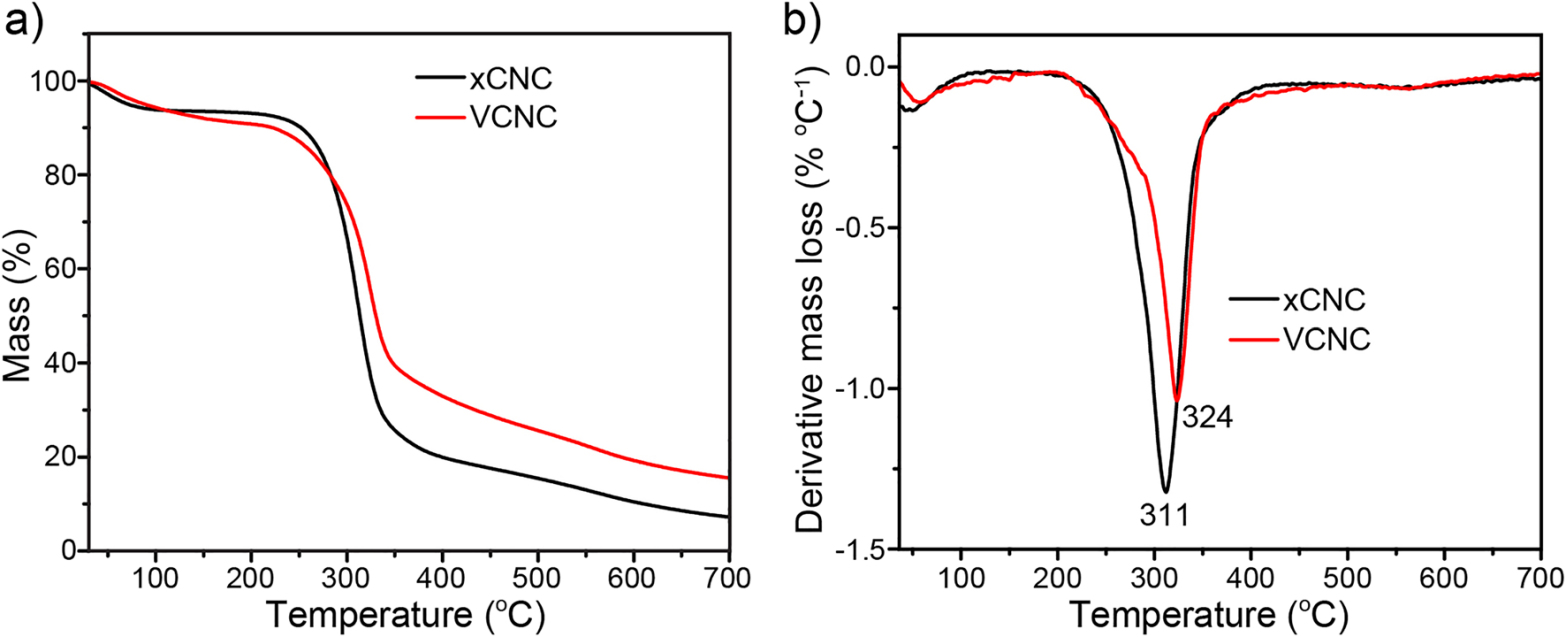
TGA results of XCNC and VCNC; (**a**) TG curves, and (**b**) DTG curves.

The TGA results of XCNC and VCNC are displayed and compared in Fig. 9. Similar TG curves were observed for both XCNC and VCNC. The initial mass loss at temperatures below 150 °C is explained by the evaporation of water adsorbed on the cellulose’s surface. Rapid mass loss at the temperature between 250 and 400 °C takes place from the decomposition of cellulose. The derivative curves indicate that the greatest decomposition rate of VCNC was found at 324 °C which is slightly greater than that of XCNC (315 °C). The results indicate the improved thermal stability of the cellulose after the TEVS treatment. It can be observed that, after the silane treatment, the carbon residue at the end of the pyrolysis increased from 8% (XCNC) to approximately 18% (VCNC). Such an increase could be attributed to the presence of SiO_2_, which was the residue from the decomposition of TEVS.

### Properties of the filled NR biocomposites

**Figure 10:**
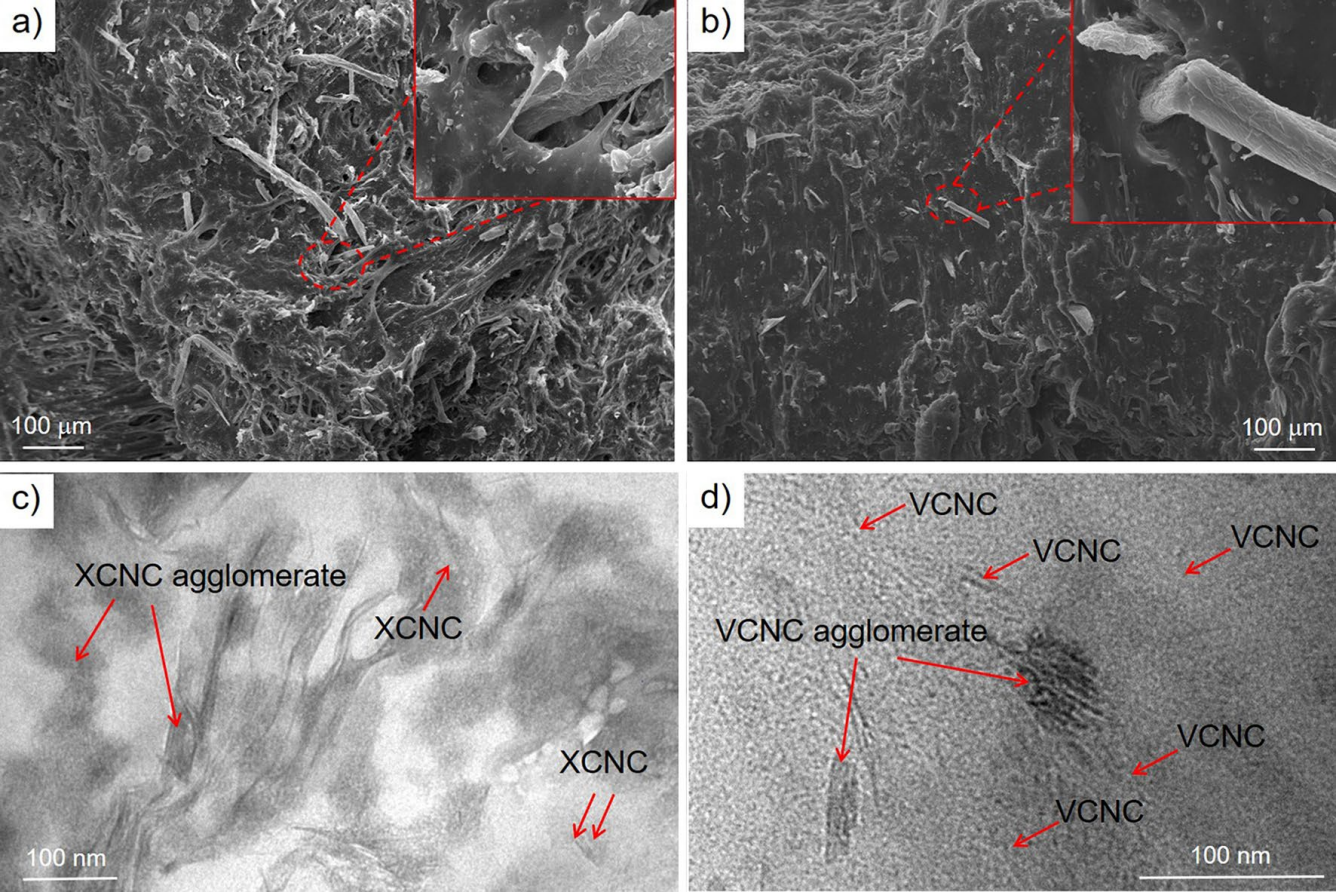
SEM and TEM images of the biocomposites; (**a**, **c**) the NR/XCNC3 and (**b**, **d**) the NR/VCNC3.

Figure 10 shows SEM and TEM images of the biocomposites filled with 3 phr of XCNC (NR/XCNC3) and VCNC (NR/VCNC3). Figure 10a exhibits the fracture surface of NR/XCNC3. Clearly, the SEM image shows a very rough surface, decorated with different particle sizes of XCNC, ranging from very fine particles to very large particles, possibly due to the filler agglomeration. The protrusion of these XCNC particles from the specimen’s surface was also observed. The SEM image taken at higher magnification (inserted Figure) shows small holes from the pulling out of the XCNC particles as well as voids at the rubber-filler interface, indicating a poor interaction between the XCNC and the rubber matrix. Compared with the NR/XCNC3, the SEM image of the NR/VCNC3, as shown in Fig. 10b, displays a smoother fracture surface with a smaller particle size of VCNC. At higher magnification (inserted Figure), the void on the rubber-filler interface is less visible, indicating the improved rubber-filler interaction after the silane treatment. Similar to the SEM results, the TEM images of NR/XCNC3 (Fig. 10c) and NR/VCNC3 (Fig. 10d), show agglomeration of the fillers in the NR matrix. Due to stronger filler-filler interaction, the filler agglomeration is more obvious in the untreated filler (XCNC). The TEM results again confirm the improved filler dispersion after the silane treatment.

**Figure 11:**
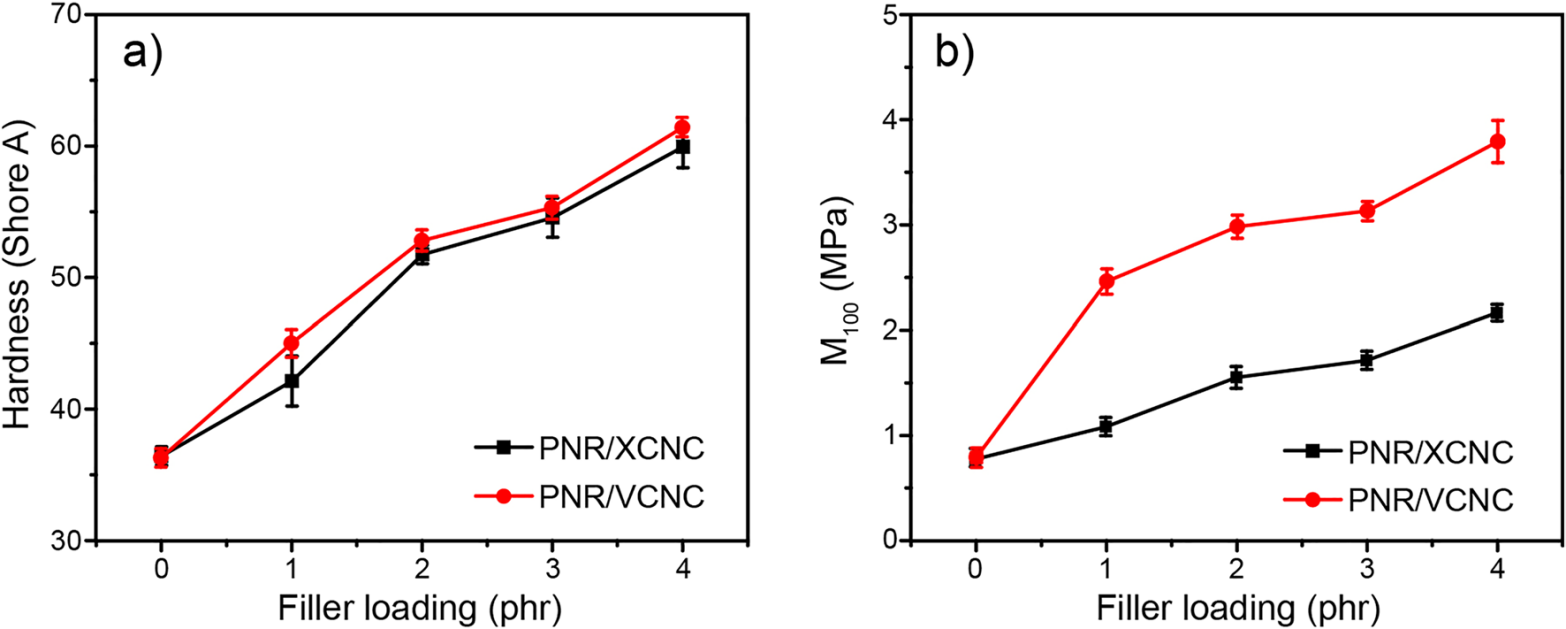
Resistance to deformation of the biocomposites; (**a**) hardness and (**b**) modulus at 100% strain (M_100_).

**Figure 12:**
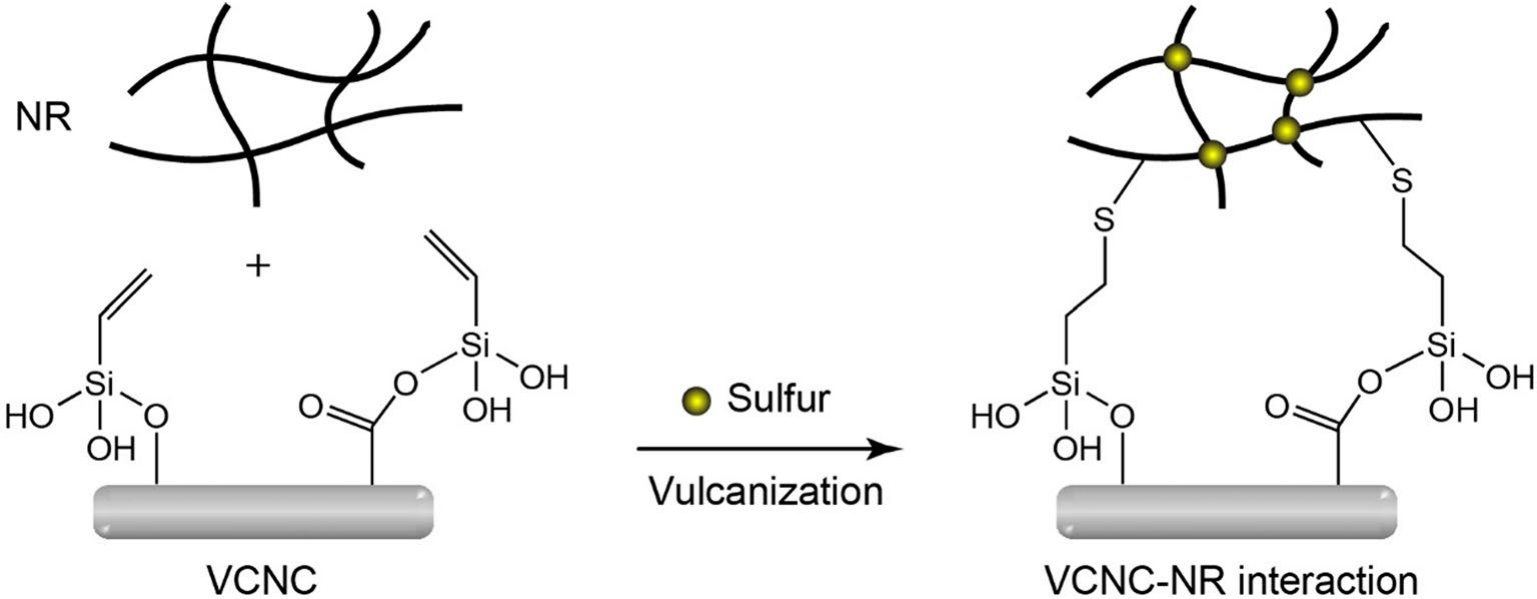
Proposed interaction between VCNC and NR.

**Figure 13:**
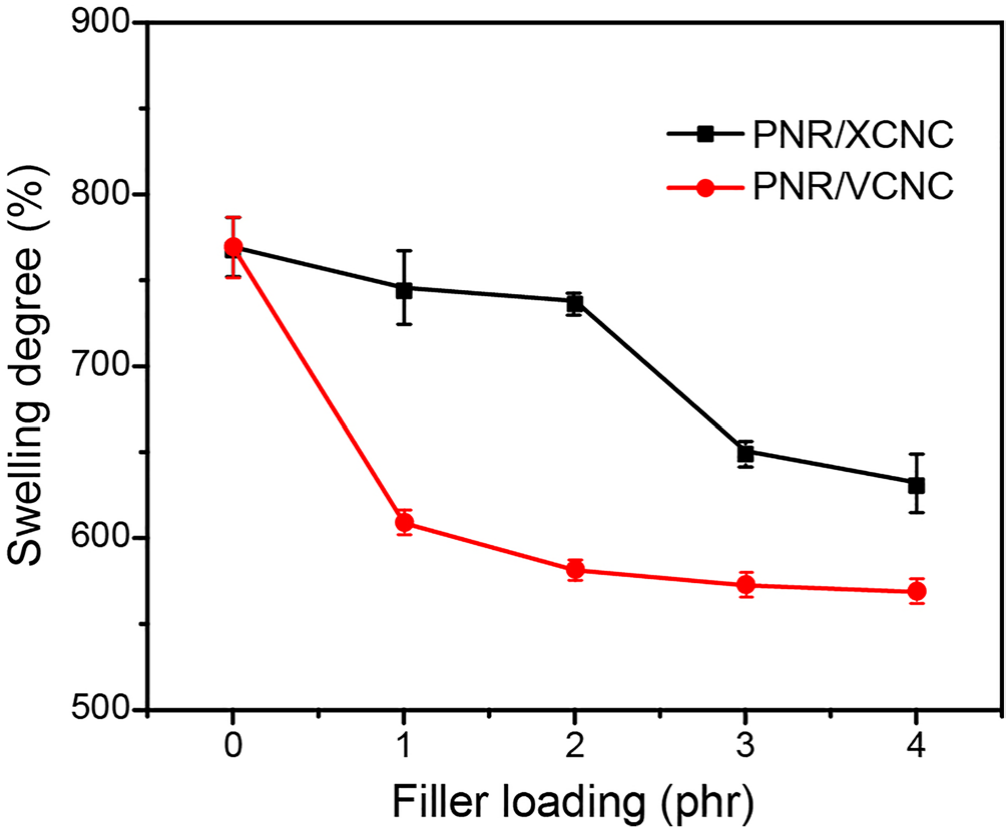
Swelling degree in toluene of the biocomposites.

**Figure 14:**
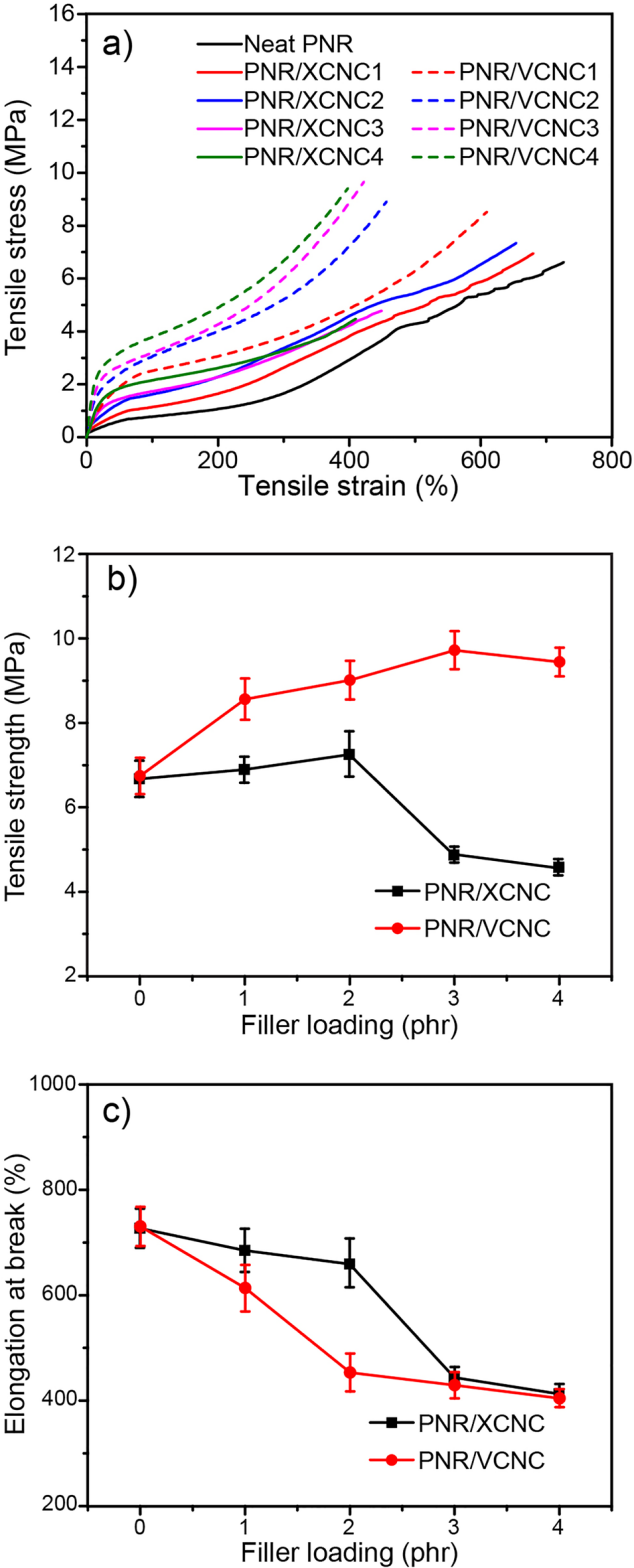
Tensile test results of the biocomposites; (**a**) stress–strain curves, (**b**) tensile strength, and (**c**) elongation at break.

Figure 11a and 11b represent hardness and tensile modulus at 100% strain (M_100_) of the biocomposites filled with different filler loadings. Due to the considerably greater stiffness of XCNC and VCNC compared to the NR matrix, the increase in XCNC or VCNC loading resulted in a reduction in the soft NR proportion, leading to continuous increases in the hardness and M_100_ of the biocomposites, widely known as the “dilution effect”. Interestingly, it can be observed that hardness increased considerably from 37 Shore A for the unfilled vulcanizate to almost 60 Shore A for the vulcanizates filled with as little as 4 phr of either XCNC or VCNC. The results reveal a very high reinforcement of both XCNC and VCNC, i.e., hardness increased by approximately 5 Shore A with the addition of every 1 phr of filler. At a given filler loading, VCNC exhibited slightly higher hardness than XCNC, possibly due to a greater magnitude of rubber-filler interaction because the TEVS grafting not only reduces the hydrophilicity of XCNC and, thus, increases compatibility with nonpolar NR matrix, but also introduces the vinyl double bonds at the chain ends that are very reactive to sulfur vulcanization, leading to strong covalent linkages as proposed in Fig. 12. This strong rubber-filler interaction greatly restricts the movement of rubber molecules, leading to greater deformation resistance, particularly at high strains. This is the reason why M_100_ of the NR/VCNC biocomposite was significantly higher than that of the NR/XCNC biocomposite when compared at any given filler loading (see Fig. 11b). As the strong rubber-filler interaction can also restrict the swelling degree of rubber in its good solvent, it is therefore found that, the swelling degree was reduced consecutively with an increase in filler loading for both XCNC and VCNC, as illustrated in Fig. 13. As expected, at the same filler loading, the NR/VCNC biocomposite had a lower swelling degree than the NR/XCNC biocomposite. Examples of stress–strain curves for the biocomposites are displayed in Fig. 14a. The average values of tensile strength and elongation at break of the biocomposites are plotted against filler loading, as displayed in Fig. 14b and 14c, respectively. In the XCNC system, the tensile strength did not change significantly with increasing XCNC loading up to 2 phr. A further increase in XCNC loading beyond 2 phr resulted in an obvious reduction in tensile strength, possibly due to the incompatibility between XCNC and NR in conjunction with the agglomeration of XCNC at high loadings. Different results were found in the VCNC system. The tensile strength of the biocomposites increased continuously with increasing VCNC loading until it reached a maximum at 3 phr, above which it reduced slightly afterwards. Due to its stronger rubber-filler interaction and better filler dispersion, VCNC gives higher tensile strength than XCNC when compared at an equal filler loading. For both filler types, elongation at break decreased continuously with increasing filler loading. This is not beyond expectation because, unlike NR, both XCNC and VCNC are not highly extendable. Due to the dilution effect, the addition of these fillers to NR reduced the elongation at break of the biocomposites. At any given filler loading, VCNC exhibited lower elongation at break than XCNC, probably due to the greater rubber-filler interaction, which provides a greater restriction of molecular movement.

## Conclusions

In this study, carboxylated cellulose nanocrystal (XCNC) was prepared from Napier grass stems via alkali treatment, bleaching, and the KMnO_4_/oxalic acid redox reaction. The removal of hemicellulose, lignin, and other non-cellulosic components from Napier grass stems was proven by TEM, SEM, FTIR, XRD, TGA and XPS. The average diameter and length of the prepared XCNC were 11.5 and 156 nm, respectively. The carboxyl content of XCNC was 1.21 mmol g^−1^. When incorporated into NR, the rubber stiffness represented in terms of modulus and hardness increased considerably with increasing XCNC loading, while tensile strength was not significantly affected up to 2 phr of XCNC, above which it tended to decline drastically due to the filler agglomeration and poor rubber-filler interaction. By using green ultrasound irradiation, the TEVS-modified XCNC (VCNC) was successfully prepared with a short reaction time and mild conditions. Due to the presence of TEVS, VCNC is less hydrophilic and, hence, more compatible with NR. In addition, the vinyl group in TEVS is highly reactive to sulfur vulcanization reaction, leading to a stronger rubber-filler interaction compared to XCNC. At the same filler loading, the NR/VCNC biocomposites therefore exhibited considerably higher tensile strength and modulus than the NR/XCNC biocomposites. The results reveal the great potential of using VCNC as a highly reinforcing filler for NR.

## Data Availability

The datasets generated and/or analysed during the current study are available at https://chem.sc.kku.ac.th/chomsri/expt-data2022.
